# Hotspots and trends of artificial intelligence in the field of cataracts: a bibliometric analysis

**DOI:** 10.1007/s10792-024-03207-5

**Published:** 2024-06-23

**Authors:** Si Chen, Li Huang, Xiaoqing Li, Qin Feng, Huilong Lu, Jing Mu

**Affiliations:** 1 Department of Ophthalmology, Jinshan Branch of Shanghai Sixth People’s Hospital, Shanghai, 201599 China; 2 Department of Ophthalmology, Shanghai Sixth People’s Hospital, Shanghai Jiao Tong University School of Medicine, Shanghai, 200235 China

**Keywords:** Artificial intelligence, Cataract, Hotspot, Bibliometric analysis, Citespace, VOSviewer

## Abstract

**Purpose:**

To analyze the hotspots and trends in artificial intelligence (AI) research in the field of cataracts.

**Methods:**

The Science Citation Index Expanded of the Web of Science Core Collection was used to collect the research literature related to AI in the field of cataracts, which was analyzed for valuable information such as years, countries/regions, journals, institutions, citations, and keywords. Visualized co-occurrence network graphs were generated through the library online analysis platform, VOSviewer, and CiteSpace tools.

**Results:**

A total of 222 relevant research articles from 41 countries were selected. Since 2019, the number of related articles has increased significantly every year. China (n = 82, 24.92%), the United States (n = 55, 16.72%) and India (n = 26, 7.90%) were the three countries with the most publications, accounting for 49.54% of the total. The Journal of Cataract and Refractive Surgery (n = 13, 5.86%) and Translational Vision Science & Technology (n = 10, 4.50%) had the most publications. Sun Yat-sen University (n = 25, 11.26%), the Chinese Academy of Sciences (n = 17, 7.66%), and Capital Medical University (n = 16, 7.21%) are the three institutions with the highest number of publications. We discovered through keyword analysis that cataract, diagnosis, imaging, classification, intraocular lens, and formula are the main topics of current study.

**Conclusions:**

This study revealed the hot spots and potential trends of AI in terms of cataract diagnosis and intraocular lens power calculation. AI will become more prevalent in the field of ophthalmology in the future.

## Background

Cataracts are common eye diseases caused by lens opacity and are one of the main causes of blindness worldwide. According to research statistics, the number of people who are blind due to cataracts accounts for one-third of the total number of blind people globally, and the number of people worldwide who are visually impaired or blind due to cataracts is as high as tens of millions [1]. There are many types of cataracts, and age-related cataracts are the most common. The incidence of age-related cataracts is positively correlated with age; women are at higher risk than men are, and the age of onset is mostly between 45 and 50 years [2–4]. Cataracts are primarily painless vision loss that can affect contrast, color perception, refractive changes, and even blindness.

In clinical practice, early cataract screening relies mainly on a slit lamp microscope to observe the lens. The diagnosis of cataracts can also rely on anterior or posterior photography to analyze the severity of the disease. For example, Hall et al. used a laser slit lamp to capture images of the anterior segment of cataract patients and performed software analysis to provide an effective and reproducible method for measuring nuclear cataracts [5]. Currently, the preferred treatment for cataracts is surgery, especially phacoemulsification cataract surgery, which has the advantages of high safety and rapid recovery [3]. Accurate intraocular lens (IOL) power calculations are very important before cataract surgery can be combined with IOL implantation and directly affect the refractive outcome of cataract surgery. At present, the most widely used IOL power calculation formulas, such as the Barrett Universal II (BUII) [6], Hoffer Q [7], Holladay 1 [8], and Sanders–Retzlaff–Kraff theories [9], primarily use biometric measurements to predict refractive results for different IOL types and powers. Moreover, these formulas, especially the BUII formula, have been proven to have good accuracy [10, 11]. Currently, there are many kinds of IOLs on the market. In addition to a single focus, there are multiple foci lenses and astigmatism-correcting lenses, which can be selected according to the different needs of patients [12].

Recently, artificial intelligence (AI) has garnered heightened attention and piqued the interest of many. As AI technology advances swiftly, major breakthroughs and opportunities have been made for scientific research, and AI is being increasingly widely used in the medical field. The global population’s aging trend is on the rise, paralleled by a growing incidence of cataract-induced visual impairments. Therefore, perfecting the diagnosis, treatment, and management of cataracts is tricky. AI brings new opportunities and challenges to solving this problem.

Bibliometrics is an interdisciplinary science that integrates mathematics, statistics and philology and can be used for quantitative evaluation and research trend analysis. VOSviewer and CiteSpace are frequently employed as graphical software tools within the realm of bibliometric studies [13, 14]. Bibliometric analysis is easy to perform, reliable, and efficient and has been widely used in the field of medical care [15, 16]. For example, through a bibliometric analysis, Chen et al. identified targeted immunotherapy as the research focus for hematological malignancies [17]. Nonetheless, the specific research focal points and future directions for AI applications in the cataract field remain uncharted. This study is a pioneering endeavor that employs bibliometric analysis to illuminate emerging trends and critical areas of focus in AI’s progress related to cataract treatment.

## Methods and materials

### Data source and research process

Data were obtained from the Science Citation Index Expanded (SCI-E) of the Web of Science Core Collection (WoSCC) on April 19, 2024. Here is the search formula: (TS = (“artificial intelligence”) OR TS = (“machine intelligence”) OR TS = (“robot*”) OR TS = (“robot technology”) OR TS = (“assistant robot”) OR TS = (“robot-assisted”) OR TS = (“computational intelligence”) OR TS = (“computer reasoning”) OR TS = (“deep learning”) OR TS = (“machine learning”) OR TS = (“neural network*”) OR TS = (“data learning”) OR TS = (“natural language processing”) OR TS = (“support vector machine*”) OR TS = (“data mining”) OR TS = (“neural network*”) OR TS = (“bayesian network*”) OR TS = (“intelligent learning”) OR TS = (“feature* learning”) OR TS = (“feature* extraction”) OR TS = (“feature* mining”) OR TS = (“feature* selection”) OR TS = (“unsupervised clustering”) OR TS = (“image* segmentation”) OR TS = (“supervised learning”) OR TS = (“semantic segmentation”) OR TS = (“deep network*”) OR TS = (“neural learning”) OR TS = (“neural nets model”) OR TS = (“graph mining”) OR TS = (“data clustering”) OR TS = (“big data”) OR TS = (“knowledge graph”)) AND (TS = (“cataract*”) OR TS = (“lens opacit*”) OR TS = (“Intraocular lens”)). The search languages were set to English and the cut-off time was set to December 31, 2023. After the above criteria were met, all the studies were individually screened and examined by two researchers. Exclude reviews, books, conference papers, and retracted publications. Sift through the remaining articles by carefully reading the title and abstract of each literature. The criteria for manual exclusion are as follows: 1) no association with AI and cataracts; 2) AI and cataracts were the main research objects. In the event of a dispute, a decision was discussed at a group meeting. The search and analysis process are depicted in Supplementary Fig. 1.

**Fig. 1:**
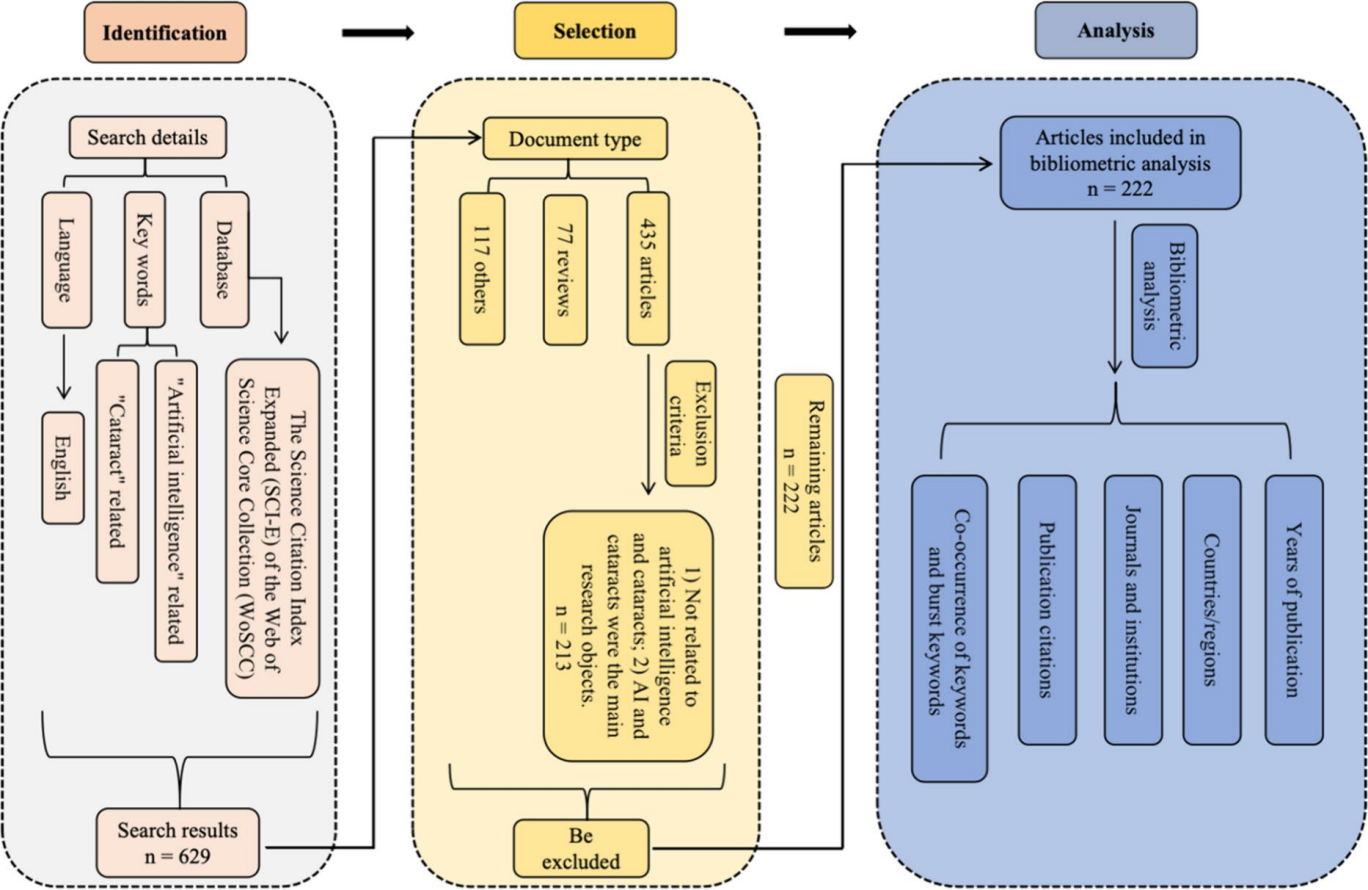
Document collection and selection process flow chart and research framework

### Bibliometric analysis

This paper employs three analytical tools to conduct a comprehensive bibliometric examination of the literature encompassing diverse facets of the subject matter. First, the library online analysis platform (https://bibliometric.com/) was used to visualize cooperative network relationships between countries/regions. Second, VOSviewer 1.6.19.0 (Leiden University, Leiden, Netherlands) was used. VOSviewer is mainly oriented to document data, and its core functions are “co-occurrence clustering” and visualization. In this study, it was used to analyze the co-occurrence network of keywords in all the documents. The different colors on the graph represent different clusters, the circles represent keywords, and the circle diameter is proportional to the frequency and correlation of occurrence. CiteSpace 6.3.1.0 (San Diego, United States) was used. CiteSpace can be used for both basic literature analysis and clustering and burst analysis. In this study, the top 20 strongest burst keywords were analyzed, and the year and intensity of the keyword bursts were determined.

## Results

### Years of publication

**Fig. 2:**
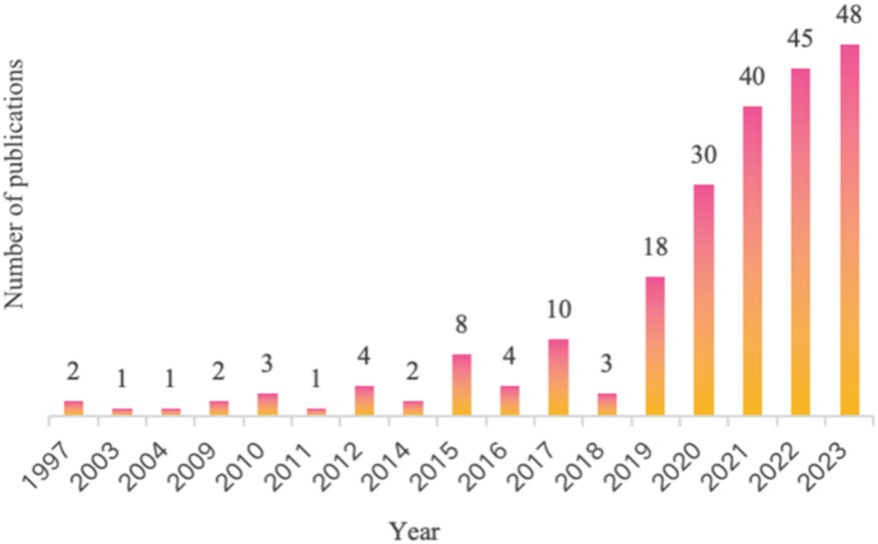
The distribution of annual publications

A total of 222 articles were obtained after screening. The popularity trend of a certain research direction can be reflected by the change in the number of published papers every year. As shown in Fig. 2, the first of these 222 articles were published in 1997. The annual publication count has seen a substantial rise, starting from just two articles in 1997 and climbing to 48 (21.62%) in the year 2023. Between 1997 and 2018, the number of articles published each year remained low, at no more than 10. However, since 2019 (18, 8.11%), the number of research publications on AI in the field of cataracts has grown rapidly, with the majority of these studies published in 2019–2023, accounting for 81.53% (181) of all publications.

### Countries/regions

**Fig. 3:**
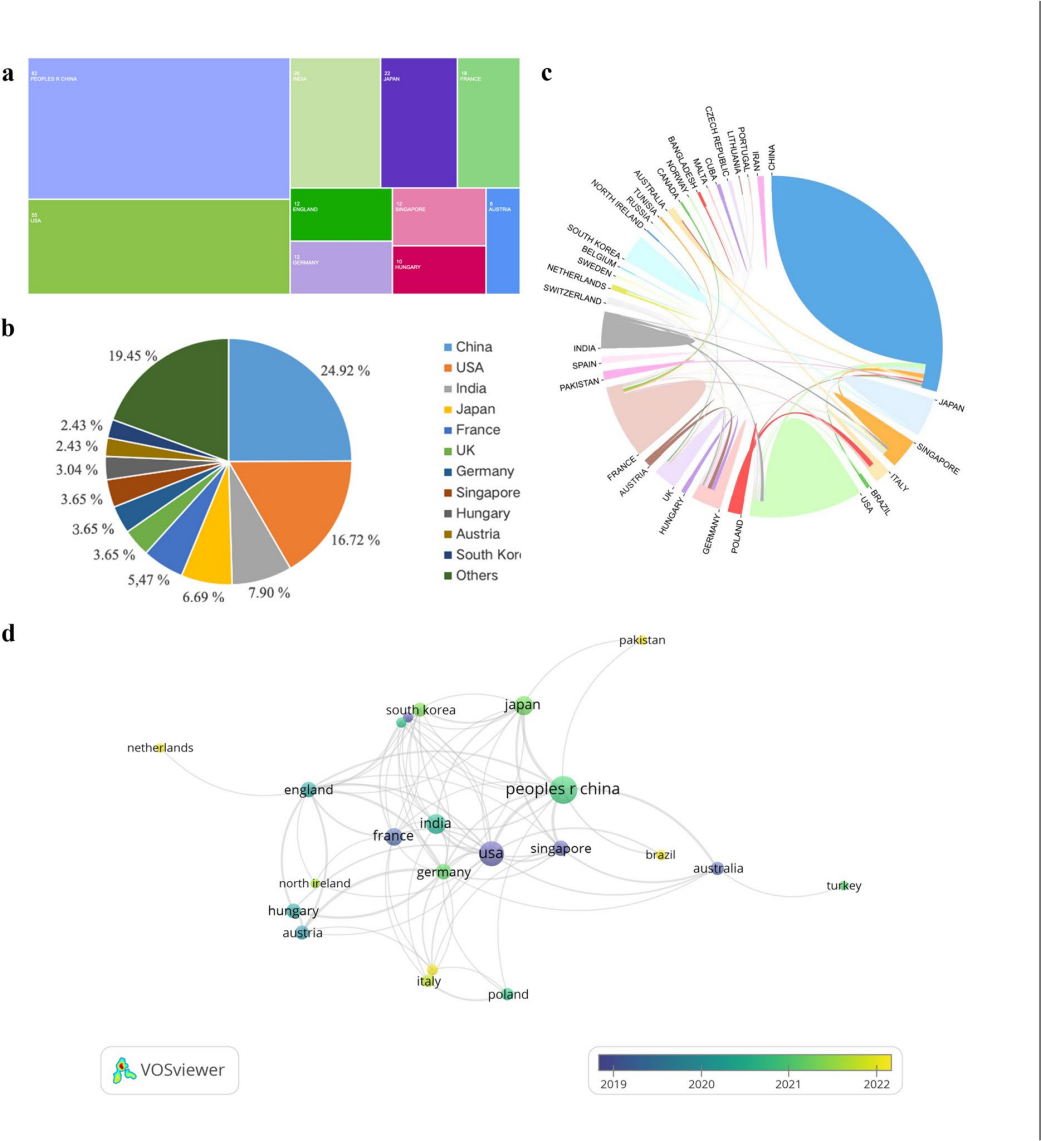
National publications overview. **a** Top 10 countries by number of publications. **b** Proportion of publications in different countries. **c** The collaboration of countries. Each country is depicted by a block of color, with larger blocks representing a greater number of publications. The line between the blocks signifies international cooperation. The thicker the line, the stronger the collaboration. **d** The map of citation relationships between countries over time

According to the statistics, the relevant literature comes from 41 countries/regions. Figure 3 shows the top 10 countries for publications in different countries (Fig. 3a) and Proportion of publications in different countries (Fig. 3b). The top 10 countries are China (n = 82, 24.92%), the United States (n = 55, 16.72%), India (n = 26, 7.90%), Japan (n = 22, 6.69%), France (n = 18, 5.47%), the United Kingdom (n = 12, 3.65%), Germany (n = 12, 3.65%), Singapore (n = 12, 3.65%), Hungary (n = 10, 3.04%), Austria (n = 8, 2.43%) and South Korea (n = 8, 2.43%). The above discussion shows that China published the most papers, followed by the United States and India. Their publications accounted for 49.54% of the total.

Cooperation between different countries/regions and institutions also reflects the global academic exchange in this field to some extent. As shown in Fig. 3c, different colors represent different countries/regions, and they are connected by lines to indicate cooperation. We found that the intersection lines between China and the United States and India are thicker than those between the other regions, indicating that the three countries are closely related to each other. Figure 3d illustrates the temporal evolution of citation connections among countries. Notably, publications originating from the United States, France, Singapore, and Austria clustered predominantly around the year 2019, whereas those from China, Japan, and Germany exhibited a concentration primarily in the vicinity of 2021.

### Journals and institutions

The journals that published these 222 articles were counted. Table 1 lists the 10 journals with the highest number of publications. Among them, the first and second place journals were from the United States: *Journal of Cataract and Refractive Surgery* (n = 13, 5.86%) and *Translational Vision Science & Technology* (n = 10, 4.50%). There were 5 journals that were in third place, all with 6 publications (2.70%), namely the British Journal of Ophthalmology, Frontiers in Medicine, Computer Methods and Programs in Biomedicine, Journal of Clinical Medicine, and Scientific Reports. As shown in Table 1, the latest impact factors for the top 10 journals ranged from 2.8 to 10.6. Seven of these journals are in Quartile 1 and three are in Quartile 2. In terms of the number of publications and influencing factors, the *Journal of Cataract and Refractive Surgery* and *Translational Vision Science & Technology* may be the most popular journals.

**Table 1:** The 10 journals with the highest number of publications

No	Journals	Publications, %	IF	Quartile in category	Countries
1	Journal of cataract and refractive surgery	13 (5.86)	2.8	Q2	USA
2	Translational vision science & technology	10 (4.50)	3.0	Q2	USA
3	British journal of ophthalmology	6 (2.70)	4.1	Q1	UK
3	Frontiers in medicine	6 (2.70)	3.9	Q2	Switzerland
3	computer methods and programs in biomedicine	6 (2.70)	6.1	Q1	Netherlands
3	Journal of clinical medicine	6 (2.70)	3.9	Q1	USA
3	Scientific reports	6 (2.70)	4.6	Q1	UK
4	Biomedical signal processing and control	5 (2.25)	5.1	Q1	UK
4	IEEE transactions on biomedical engineering	5 (2.25)	4.6	Q1	USA
4	IEEE transactions on medical imaging	5 (2.25)	10.6	Q1	USA

Next, we analyzed the institutions that published the literature. Table 2 lists the top 9 institutions with the highest number of publications. We found that the top 3 institutions were all from China, namely Sun Yat-sen University (n = 25, 11.26%), the Chinese Academy of Sciences (n = 17, 7.66%), and Capital Medical University (n = 16, 7.21%). It is worth noting that China has the largest number of publications, and institutions from China also rank very high. The United States is second in terms of the number of publications, but its organizations do not rank high.

**Table 2:** Top 9 institutions with the highest number of publications

No	Institutions	Publications, %	Countries
1	Sun Yat-sen university	25 (11.26)	China
2	Chinese academy of sciences	17 (7.66)	China
3	Capital medical university	16 (7.21)	China
4	Institut National de la Sante et de la Recherche Medicale, INSERM	9 (4.05)	France
5	Southern university of science technology	9 (4.05)	China
6	Fudan university	8 (3.60)	China
7	Johns Hopkins university	8 (3.60)	USA
8	National university of Singapore	8 (3.60)	Singapore
9	Singapore national Eye centre	8 (3.60)	Singapore

### Publication citations

The total citations of articles reflect not only the recognition but also the trends and hotspots of a certain research field to a certain extent. Table 3 lists the top 11 most cited papers on the application of AI in the field of cataracts, from highest to lowest according to the total local citation score (TLCS), including publication year and journal. The main focus of the 11 related articles was on AI-based cataract diagnosis and management systems. This may be the potential trend and hot spot of AI in the field of cataract research.

**Table 3:** Top 11 most cited papers on the application of AI in the field of cataracts.

No	Title	Year	Journal	TLCS
1	An artificial intelligence platform for the multihospital collaborative management of congenital cataracts	2017	Nature Biomedical Engineering	193
2	Automatic Feature Learning to Grade Nuclear Cataracts Based on Deep Learning	2015	IEEE Transactions on Biomedical Engineering	131
3	Endophthalmitis after Cataract Surgery in the United States A Report from the Intelligent Research in Sight Registry, 2013–2017	2020	Ophthalmology	98
4	Exploiting ensemble learning for automatic cataract detection and grading	2016	Computer Methods and Programs in Biomedicine	97
5	Diagnostic Efficacy and Therapeutic Decision-making Capacity of an Artificial Intelligence Platform for Childhood Cataracts in Eye Clinics: A Multicentre Randomized Controlled Trial	2019	eClinicalMedicine	93
6	Importance of multi-modal approaches to effectively identify cataract cases from electronic health records	2012	Journal of the American Medical Informatics Association	86
7	A Framework for the recognition of high-level surgical tasks from video images for cataract surgeries	2012	IEEE Transactions on Biomedical Engineering	80
8	A computer-aided healthcare system for cataract classification and grading based on fundus image analysis	2015	Computers in Industry	74
9	A Hybrid global–local representation CNN model for automatic cataract grading	2020	IEEE Journal of Biomedical and Health Informatics	66
10	Universal artificial intelligence platform for collaborative management of cataracts	2019	Journal of Ophthalmology	64
10	A computer-aided diagnosis system of nuclear cataract	2010	IEEE Transactions on Biomedical Engineering	64

### Co-occurrence of keywords and burst keywords

Keywords are very effective at researching potential hotspots and trends in an academic field. Among the 222 articles, 124 keywords that appeared at least seven times were analyzed. As shown in Table 4, the top 20 keywords that appear most frequently in this paper were listed. The top 20 keywords were cataract (n = 109), eye (n = 96), patient (n = 95), image (n = 65), network (n = 62), system (n = 54), intraocular lens (n = 52), paper (n = 52), artificial intelligence (n = 21), area (n = 49), IOL (n = 48), feature (n = 46), detection (n = 45), detection (n = 45), classification (n = 40), diagnosis (n = 40), formula (n = 37), level (n = 31), deep learning (n = 30), error (n = 30), ophthalmologist (n = 29). Moreover, Table 4 shows that the occurrence frequency of keywords is positively correlated with the total connection strength; that is, the greater the occurrence frequency is, the greater the corresponding total connection strength.

**Table 4:** The top 10 most frequent keywords

No	Keywords	Occurrences	Total link strength
1	Cataract	109	1293
2	Eye	96	1126
3	Patient	95	1135
4	Image	65	873
5	Network	62	726
6	System	54	617
7	Intraocular lens	52	624
8	Paper	52	659
9	Artificial intelligence	50	623
10	Area	49	532
11	Iol	48	625
12	Feature	46	691
13	Detection	45	604
14	Classification	40	566
15	Diagnosis	40	543
16	Formula	37	572
17	Level	31	363
18	Deep learning	30	358
19	Error	30	357
20	Ophthalmologist	29	373

**Fig. 4:**
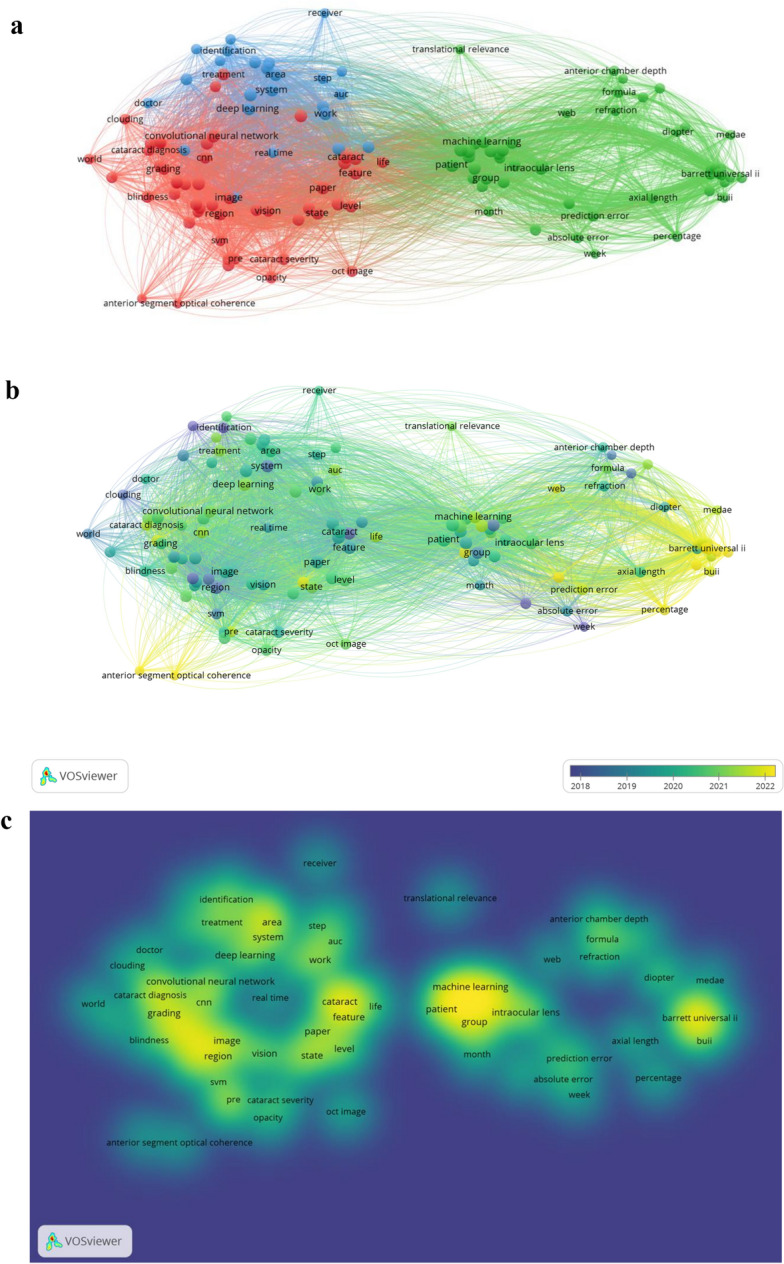
Visualization of keyword analysis. **a** Co-occurrence map of keywords. **b** Overlay map of co-occurrence keywords. The nodes indicated with purple color represent earlier-appearing keywords, while the keywords marked with yellow color show current research concerns. **c** Density visualization of keywords. Circle size, and yellow opacity are correlated with higher co-occurrence frequencies

To more intuitively reflect the research trends and hotspots of AI in the field of cataracts, we created a keyword co-occurrence network map using VOSviewer software, where the minimum number of keyword occurrences was 7 (Fig. 4a). The dimensions of nodes and words mirror the frequency with which they co-occur, while the links between them denote these co-occurrences. Utilizing the strength of item co-occurrence associations, the network is segmented into distinct clusters, each color-coded to represent its unique community. These clusters are represented mainly in red, green and blue. The content represented by the three clusters clearly shows the developmental trend of AI applications in cataract research. The red cluster was the most significant, and primarily related to the diagnosis of cataracts through cataracts, image, network, feature, classification, diagnosis, cataract severity, blindness, grading and opacity. The green cluster indicates the IOL power calculations, such as patient, intraocular lens, formula, prediction error, axial length, machine learning, refraction percentage and barrett universal ii. The key words prominent in the blue cluster were doctor, work, deep learning, real time, step, identification system and area.

Figure 4b summarizes the visual map of keywords in terms of time. These keywords were from 2018 to 2022. Nodes highlighted in yellow denote the most recent average year of publication activity, suggesting these may constitute focal areas in the diagnosis and treatment of cataracts. The keywords were machine learning, cataract, image, system, diagnosis, group and cataract severity, between 2018 and 2020. These central keywords predominantly revolve around advancements in cataract diagnosis. Between 2021 and 2022, the keywords were mainly axial length, prediction error, intraocular lens, barrett universal ii and percentage, indicating a focus on IOL-related. Figure 4c shows a density visualization of keywords. The dimensions of the circles, coupled with their yellow translucency, correlate directly with heightened frequencies of co-occurrence. Keywords that occurred more frequently were cataract, feature, image, grading, diagnosis, group, patient, intraocular lens and barrett universal ii.

**Fig. 5:**
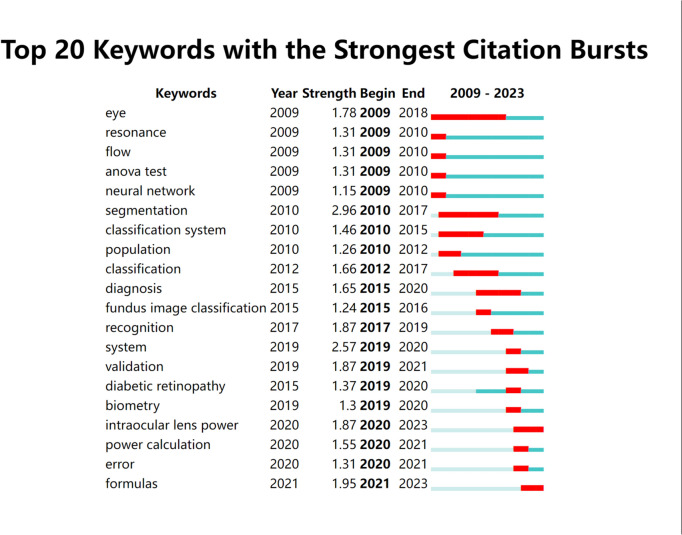
Top 20 keywords with the strongest citation bursts based on CiteSpace

Strongly cited explosive keywords are keywords that have been widely cited within a short period of time and are considered important research hotspots and future trends. This study utilized the breakout word detection function of CiteSpace to obtain the top 20 keywords with strong citation bursts from 2009 to 2023 (Fig. 5). The left side of Fig. 5 shows the top 20 most highly cited explosive keywords, the year of occurrence, and the intensity of citation; the thick red line on the right represents the time period of the outbreak of this keyword in the corresponding year. The three keywords with the highest burst intensities were segmentation (2.96), system (2.57) and formulas (1.95). In addition, the keyword with the longest outburst time was eye (9 years). Moreover, the keywords associated with the most recent outbreak included intraocular lens power and formulas. These burst keywords also reflect the research trend of AI in the field of cataracts.

## Discussion

Bibliometrics analysis was performed to examine the research hotspots and trends of AI in the field of cataracts from a variety of angles, including years, countries/regions, journals, institutions, publication citations, and keywords. In this study, through bibliometric analysis, we found that cataract diagnosis and IOL power calculation are potential research trends and hotspots of AI in the field of cataracts.

### Years of publication

In this study, a total of 222 publications were selected that fit the topic. The number of papers in the topic of cataracts pertaining to AI has slowly increased since 1997. 2019 marked a clear turning point, since there was substantial increase from 2019 to 2021. The quick development of AI technology and the world economy may be connected to this. In recent years, although the number of relevant studies published has increased annually, the number of AI papers in the field of cataracts accounts for only a small part of the entire field of ophthalmology. For example, according to Zhao et al. the number of papers published by AI in the field of eye disease diagnosis between 2012 and 2021 is as high as 1,498 [18].In fact, in the field of ophthalmology, many scholars around the world have used AI to help treat various eye diseases. For example, Varun Gulshan et al. created an algorithm based on deep machine learning to automatically detect and evaluate diabetic retinopathy and diabetic macular edema in retinal fundus photographs with high sensitivity and specificity [19]. These suggest that AI will continue to receive attention in the field of cataracts.

### Countrues/regions, journals, and institutions

It seems that Chinese scholars were very enthusiastic about AI research in cataracts, as evidenced by the number of papers and top-ranked institutions in terms of publications. China’s publications were concentrated in 2021 when viewed from a temporal viewpoint, which was also indicative of the country’s recent rapid advancement in AI-related technology. Furthermore, the United States ranks second only to China in terms of the quantity of relevant articles. The two journals with the highest number of publications were both from the United States. This shows that American journals are more globally recognized. Interestingly, the year of publication concentration in the United States was 2019, earlier than China. The two countries that contribute most to this topic are China and the United States, and there is close cooperation between the two. This outcome is consistent with many other bibliometric analyses on AI-related topics [20, 21].

### Publication citations

Consistent with our findings, six of the top 11 most cited papers dealt with cataract diagnosis. Gao et al. for instance, employed support vector regression to automatically features learning to grade cataracts [22]. Biomedical journals are known to emphasize high quality related research, as evidenced by the fact that six out of the top 11 most cited publications were published in such journals. This serves as a valuable guidepost for scholars aspiring to publish high-caliber articles on comparable subjects, offering them a potent benchmark for journal selection.

### Co-occurrence of keywords and burst keywords

By meticulously analyzing the keywords, our study uncovered the prevailing focal points and emerging trends in AI research within the realm of cataract investigations. First, the AI can help assess the presence and severity of cataracts. For example, in 2015, Gao et al. extracted the features of slit lamp images of a large number of cataract patients through deep learning methods to automatically grade the severity of nuclear cataracts [22]. Zhang et al. also used deep learning to automatically diagnose and grade cataracts based on patient fundus images, with an accuracy of more than 92.66% [23]. However, whether slit lamp images or fundus images are used for deep learning is affected by image quality, noise, or the optical environment. To solve this problem, researchers have developed and verified different effective anti-interference and antinoise AI cataract diagnosis models to improve accuracy [24, 25]. In addition, Wu et al. explored a universal AI platform and a multilevel referral model that significantly improved the diagnostic performance and service efficiency of cataracts [26]. Additionally, a cataract recognition and characterization algorithm based on electronic health records was successfully developed and validated by Peissig et al. with a positive predictive value of > 95%, and it significantly increased the rate of cataract detection [27]. The AI-based cataract diagnosis model greatly improved the diagnostic efficiency and accuracy for cataract patients, helping improve the early screening, diagnosis, and treatment efficiency of cataract patients, especially in economically underdeveloped areas or areas with a shortage of ophthalmologists.

The pivotal contribution of AI in the domain of cataracts is also evident in the significant enhancements it has brought to the methods of calculating IOL power. For instance, González et al. developed a novel Karmona model for predictive IOL power calculation based on machine learning techniques with good accuracy [28]. Kenny et al. compared the prediction accuracy of the AI-based IOL power calculation formula with that of other traditional formulas in a multicenter retrospective case series study. The authors found that using segmented axial length in all the formulas did not improve refractive prediction. However, the mean absolute error and root mean square absolute error of the ZEISS AI IOL Calculator were smaller than those of Barrett, Pearl-DGS, and Kane’s formulas [29]. Similarly, several new IOL power calculation models developed using AI-based methods have been shown to produce significantly more accurate predictions than does another non-AI formula (BUII), which is generally considered the most accurate [30, 31]. The goal of treating cataracts has evolved from just correcting visual impairment to include improving postoperative visual comfort as living standards rise. The difficulty of creating more accurate and customized AI-driven IOL power calculation models appropriate for widespread clinical use is increased by these rising demands.

## Limitations

While bibliometric analysis objectively, systematically, and quantitatively examines all invaluable insights within the targeted literature, it is imperative to recognize the constraints of our investigation. In summary, three main limitations are evident. Firstly, the study confines its search to the SCI-E database within the WoSCC, acknowledged as a preeminent global citation index. Nonetheless, the scarcity of data pertinent to our subject matter restricts the breadth of our bibliometric exploration, thereby imposing limitations on the comprehensiveness of our findings. Secondly, by restricting our search parameters to English-language articles, and employing a manual screening process conducted by researchers to exclude irrelevant content, we inevitably risk omitting valuable research presented in other languages or formats beyond traditional articles. This human-mediated curation, while careful, may have been influenced by the researchers’ expertise boundaries, potentially leading to the exclusion of pertinent studies. Lastly, our research hinged on keyword extraction and analysis, centering on “AI” and “cataract”. The boundaries imposed by our search formulation or the specificity of our research theme might have precluded a comprehensive capture of all pertinent literature spanning diverse facets of the topic. Furthermore, during keyword analysis, recurrent generic terms such as cataract, artificial intelligence, system, outcomes, deep learning, and machine learning dominated the high-frequency results, which, though indicative, did not necessarily enrich our analytical insights with nuanced information. Consequently, future efforts aspire to broaden the data sources, refine keyword precision, and elevate the interdisciplinary expertise of our research team to augment the depth and reach of our analyses.

## Conclusion

This paper reveals the research hotspots and potential trends of AI in terms of cataract diagnosis and IOL power calculation. AI-based models have great potential in all aspects of cataract diagnosis and treatment, but the effectiveness and safety of AI need to be proven with a large amount of data over a long period of time. This study will help relevant researchers, clinicians, and government agencies better grasp the cutting-edge trends of AI in the field of cataracts and better integrate AI into clinical practice in the future for the benefit of humanity.

## Data Availability

All relevant data are included in the papers and its Supporting Information files. Contact to Dr. Jing Mu (jingm79@163.com) for additional information regarding data access.
